# Pharmacological and genetic targeting of 5-lipoxygenase interrupts c-Myc oncogenic signaling and kills enzalutamide-resistant prostate cancer cells via apoptosis

**DOI:** 10.1038/s41598-020-62845-8

**Published:** 2020-04-20

**Authors:** Jitender Monga, Dhatchayini Subramani, Ajay Bharathan, Jagadananda Ghosh

**Affiliations:** 1 Vattikuti Urology Institute, Henry Ford Health System, Detroit, MI 48202 United States; 2 Henry Ford Cancer Institute, Henry Ford Health System, Detroit, MI 48202 United States

**Keywords:** Prostate cancer, Prostate cancer, Prostate, Prostate

## Abstract

Much of the morbidity and mortality due to prostate cancer happen because of castration-resistant prostate cancer (CRPC) which invariably develops after anti-androgenic therapy. FDA-approved enzalutamide is commonly prescribed for CRPC which works by blocking androgen receptor function. However, even after initial good response, enzalutamide-resistant prostate cancer (ERPC) develops which eventually leads to widespread metastasis. Management of ERPC is extremely difficult because available therapeutic regimen cannot effectively kill and eliminate ERPC cells. Though the mechanism behind enzalutamide-resistance is not properly understood, over-activation of c-Myc has been found to be a common event which plays an important role in the maintenance and progression of ERPC phenotype. However, direct-targeting of c-Myc poses special problem because of its non-enzymatic nature and certain amount of c-Myc activity is needed by non-cancer cells as well. Thus, c-Myc has emerged as an elusive target which needs to be managed by novel agents and strategies in a cancer-specific way. We investigated the effects of pharmacological and genetic inhibition of 5-lipoxygenase (5-Lox) on cell proliferation, apoptosis and invasive potential of enzalutamide-resistant prostate cancer cells. Transcriptional activity of c-Myc was analyzed by DNA-binding, luciferase-assays, and expression of c-Myc-target genes. We found that 5-Lox regulates c-Myc signaling in enzalutamide-resistant prostate cancer cells and inhibition of 5-Lox by Quiflapon/MK591 or shRNA interrupts oncogenic c-Myc signaling and kills ERPC cells by triggering caspase-mediated apoptosis. Interestingly, MK591 does not affect normal, non-cancer cells in the same experimental conditions. Our findings indicate that inhibition of 5-Lox may emerge as a promising new approach to effectively kill ERPC cells sparing normal cells and suggest that development of a long-term curative therapy of prostate cancer may be possible by killing and eliminating ERPC cells with suitable 5-Lox-inhibitors.

## Introduction

Prostate cancer is the most prevalent form of malignancy and a leading cause of cancer-related deaths in American men taking thousands of lives every year^1^. Much of the morbidity and mortality due to prostate cancer is caused by castration-resistant prostate cancer (CRPC), which invariably develops after initial good response with androgen-deprivation therapies (ADT). Enzalutamide, an FDA-approved inhibitor of androgen receptor function, is commonly prescribed to treat castration-resistant prostate cancer^2–5^. While Enzalutamide improves survival and quality of life of patients with castrate-resistant disease, which highlights the benefit of targeting the AR axis in CRPC, enzalutamide-resistant prostate cancer (ERPC) almost always develops which leads to widespread metastatic disease eventually bringing demise to prostate cancer patients^4–6^. ERPC is not curable because currently available therapeutic regimen cannot effectively kill and eliminate ERPC cells. Thus, ERPC is considered as a death threat to prostate cancer patients, signifying an urgent need to develop new, more effective strategies to overcome enzalutamide-resistance and save thousands of men’s lives that are lost every year.

Phenotypic transition of androgen-dependent prostate cancer to androgen independence presumably involves an array of complex processes with both selection of pre-existing clones of androgen-independent cells, as well as the *de novo* development plus selection of new clones of cells with altered genetic events. A number of genetic changes have been identified and characterized which play roles in Enzalutamide-resistance. This list includes reactivation of the AR signaling (via AR gene amplification or mutation or generation of splice variants), activation of AR bypass mechanism (via induction of glucocorticoid receptor), or development of AR-independent mechanisms which help the cancer cells to survive and grow in an environment deficient of androgenic signaling^7^. Some similarities exist in mechanisms contributing to resistance to various inhibitors of androgenic signaling. One such molecular mechanism for evolution of Enzalutamide-resistant prostate cancer is over-activation of the Myc oncogene. Over-activity of c-Myc is one of the most frequent genetic event observed to be associated with androgen-resistant prostate tumors, and experimentally c-Myc was characterized to promote androgen-independent growth of prostate cancer cells^8–10^. A common amplicon has been detected during the conversion to androgen-independent prostate cancer in a short region spanning chromosome 8q which also contains the c-Myc oncogene, and in more than 70% of clinical androgen-independent prostate tumor samples, amplification of the c-Myc gene has been found by fluorescence *in situ* hybridization^11,12^. Moreover, an increase in c-Myc gene amplification was repeatedly observed after treatment with inhibitors of androgenic-signaling^13,14^, and Bernard *et al*. observed that overexpression of c-Myc is sufficient to induce androgen-independent growth of LNCaP cells treated with the anti-androgen, casodex^8^. Thus, over-activity of c-Myc needs to be properly controlled to inhibit growth of prostate cancer, especially which are resistant to anti-androgenic therapies. However, because of its non-enzymatic nature and absence of any deep pocket, specific targeting of c-Myc transcriptional activity by blocking its protein-protein or protein-DNA interaction, is extremely difficult^15,16^. Thus, in spite of being recognized as a *bona fide* promoter of anti-androgenic therapy-resistant prostate cancer, Myc remained as an elusive molecular target for developing strategies to overcome Enzalutamide-resistance.

Recently we reported that inhibition of arachidonate-5-lipoxygenase (5-Lox) by gene-targeting or by chemical inhibitors down-regulates expression and function of c-Myc selectively in cancer cells, but spares c-Myc activity in normal, non-cancer cells^17,18^. Since c-Myc plays an important role in the transition from androgen-dependent prostate cancer to the androgen-refractory phenotype, we asked the question whether 5-Lox regulates c-Myc signaling and the viability of prostate cancer cells when they become resistant to enzalutamide therapy. We were especially interested in ERPC because enzalutamide, which is prescribed post-docetaxel failure, extends life-span, but no other treatment option remains when enzalutamide-resistance develops, and currently most of the lives lost due to prostate cancer is because of the development of ERPC^19,20^. We addressed a possible role of 5-Lox in the survival of the ERPC cells using the MR49F and LNCaP-ENR human prostate cancer cells which were derived from the androgen-sensitive LNCaP cells after multiple passaging through castrated hosts, and/or maintaining in long-term cultures in the presence of serum-equivalent doses (10–30 μM) of enzalutamide^21^. We found that 5-Lox is heavily expressed in ERPC cells, and inhibition of 5-Lox by specific chemical inhibitor (e.g., MK591) or shRNA downregulates c-Myc and targets, and kills ERPC cells via caspase-mediated apoptosis. We also found that in contrast to the ERPC cells which express high levels of 5-Lox, the expression of 5-Lox in normal, non-cancer cells (e.g., astrocytes, human fore-skin fibroblasts) is undetectable, and that the normal cells are not affected by 5-Lox inhibition. These novel findings document a unique regulation of c-Myc oncogene and the survival of ERPC cells by 5-Lox-mediated signaling and suggest that targeting 5-Lox may turn out to be an excellent approach to effectively and selectively eliminate the ERPC cells via induction of apoptosis, which may help establish a new foundation to overcome ERPC and prevent prostate cancer recurrence.

## Results

### Enzalutamide triggers upregulation of c-Myc in androgen-sensitive prostate cancer cells

**Figure 1:**
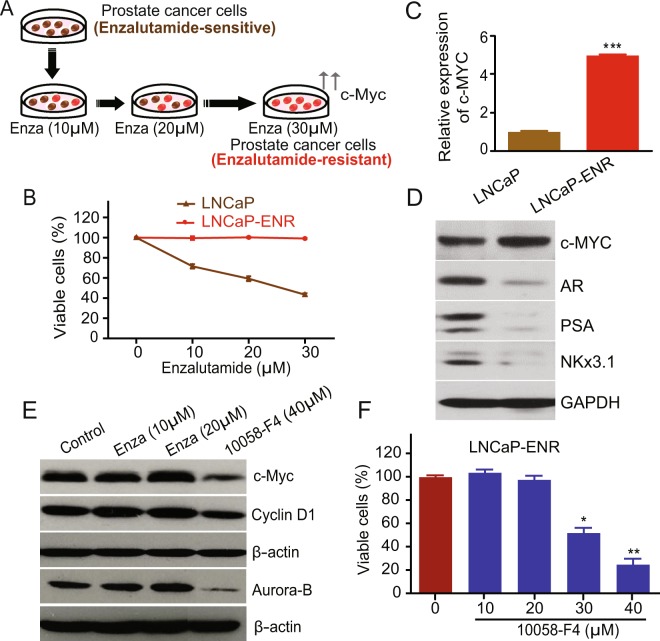
Upregulation of c-Myc in prostate cancer cells by Enzalutamide treatment. (**A**) Human LNCaP prostate cancer cells were treated with increasing doses of Enzalutamide for 6 months to generate the LNCaP-ENR cell line. (**B**) Effect of enzalutamide on cell viability of the parental LNCaP and the derived LNCaP-ENR cells were tested by plating 3,000 cells per well in 96 well plates and treating with doses of enzalutamide. Plates were incubated further for 72 hours and cell viability was measured by MTS/PES assay. (**C**) Levels of c-Myc mRNA in LNCaP and LNCaP-ENR cells were measured by RT-PCR. (**D**) Protein levels of c-Myc, AR, PSA and NKX3.1 were detected by Western blot. (**E**) LNCaP cells were treated with enzalutamide in short-term culture (for 48 hours) and then c-Myc and target proteins in whole cell lysates were detected by Western blot using beta-actin as loading control. The full-length blots are presented in Supplementary Fig. 1. (**F**) LNCaP-ENR cells were treated with a c-Myc inhibitor (10056-F4) as indicated and cell viability was measured by MTS/PES assay. *Note:* Results are presented as mean values of each data point ± standard error (S.E.) and the significance was consistently determined as *p < 0.05, **p < 0.005, ***p < 0.0005, where applicable.

To investigate the role of 5-Lox in enzalutamide resistance, we generated a prostate cancer cell line model with acquired enzalutamide resistance. For this, wild-type prostate cancer cells, LNCaP, were cultured with increasing concentrations of enzalutamide over a period of approximately 6 months to generate LNCaP-ENR cells (Fig. 1A). The resistance status at each dose of enzalutamide was determined by MTS cytotoxicity assay (Fig. 1B). Also, both the mRNA and protein levels of c-Myc are increased in LNCaP-ENR cells compared to parental LNCaP cells (Fig. 1C,D). However, protein levels of AR and its targets (PSA, NKX3.1) were found to be decreased, suggesting that LNCaP-ENR cells do not develop reactivation of the AR signaling (Fig. 1D). Over-activation of c-Myc has been repeatedly found in castration-resistant prostate tumors and characterized to play an important role in the maintenance of CRPC phenotype^10,14,22^. We found that when the androgen-sensitive LNCaP prostate cancer cells were treated with enzalutamide in short-term culture, there was an increase in the protein level of c-Myc and its targets (Fig. 1E). Moreover, we found that treatment with 10058-F4, an inhibitor of c-Myc transcriptional activity, decreased the viability of enzalutamide-resistant (LNCaP-ENR) prostate cancer cells, documenting that the c-Myc activity plays an important role in the survival of ERPC cells (Fig. 1F). These findings corroborate previous studies which suggested that c-Myc plays a crucial role in the development and maintenance of CRPC phenotype by promoting cell proliferation and preventing cell death.

### Enzalutamide-resistant prostate cancer cells express high levels of 5-Lox, and inhibition of 5-Lox interrupts c-Myc oncogenic signaling

**Figure 2:**
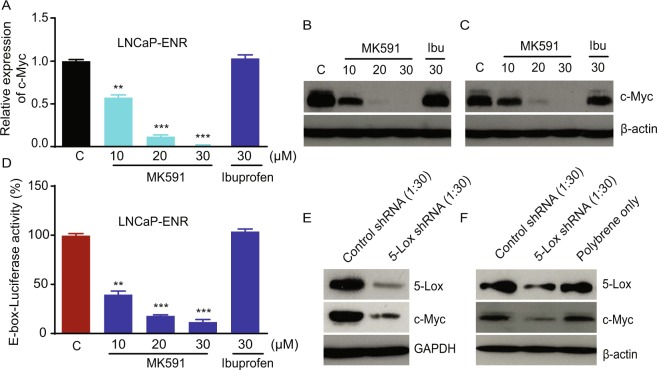
Decrease in c-Myc mRNA and protein levels by chemical inhibitor and shRNA of 5-Lox. (**A**–**C**) LNCaP-ENR and MR49F prostate cancer cells were plated in 60 mm diameter plates (~300,000 cells per plate) for 48 hours and treated with varying doses of Enzalutamide for 24 hours. Level of mRNA was detected in treated and untreated cells by RT-PCR. Cell lysate proteins were separated in 12% SDS-PAGE and c-Myc protein level was detected by Western blot. (**D**) LNCaP-ENR cells transfected with E-box-luciferase constructs were plated and treated with doses of MK591. After 24 hours, luciferase activity was measured in a Perkin-Elmer luminescence plate reader using stable-Glo substrate from Promega Corp (Madison, WI). (**E**,**F**) LNCaP-ENR and MR49F cells were treated with lentiviral shRNA against 5-Lox (or control shRNA) for 96 hours. Protein levels of 5-Lox and c-Myc were detected by Western blot. The full-length blots are presented in Supplementary Fig. 1. *Note:* Results are presented as mean values of each data point ± standard error (S.E.). Significance was determined as **p < 0.005, ***p < 0.0005, where applicable.

Though c-Myc has emerged as a promising, stand-alone molecular target for management of ERPC based on its critical role in the promotion and maintenance of ERPC cell survival and proliferation, effective measures to selectively downregulate c-Myc in ERPC cells, while sparing non-cancer cells, is extremely challenging. Interestingly, we found that MK591, a specific, bio-available inhibitor of 5-Lox activity^23–27^, dramatically decreased the mRNA and protein level as well as the transcriptional activity of c-Myc in ERPC cells in a clear dose-dependent manner (Fig. 2A–D). Moreover, we found similar downregulation of c-Myc protein levels in ERPC cells when the 5-Lox gene expression was targeted by shRNA (Fig. 2E,F). These findings indicate that the 5-Lox activity plays an important role in regulating the expression and function of c-Myc in ERPC cells. Note: Ibuprofen (an inhibitor of cyclooxygenase) was used in parallel experiments which did not show any noticeable inhibition of c-Myc, suggesting that the effect of 5-Lox inhibition on c-Myc is highly selective.

### Inhibition of 5-Lox decreases the viability of Enzalutamide-resistant prostate cancer cells, but not of normal, non-cancer cells

**Figure 3:**
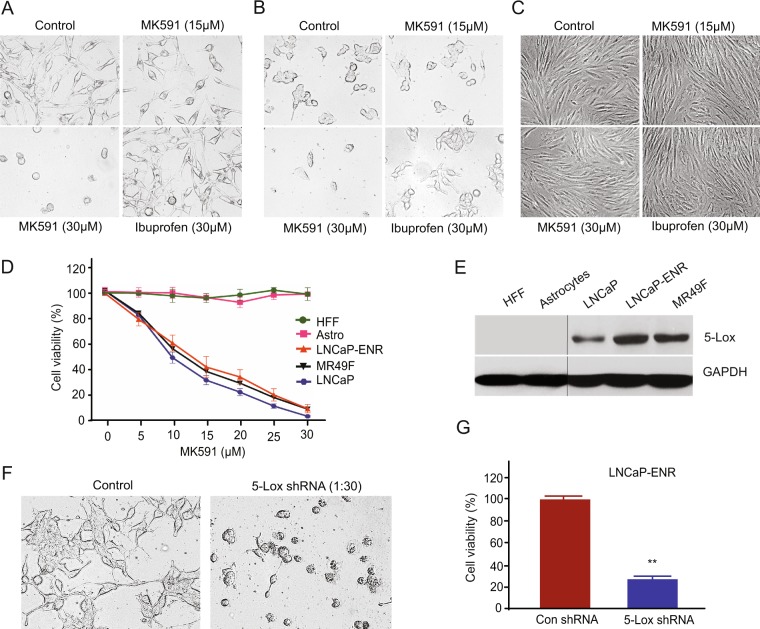
Selective effects of 5-Lox inhibition on Enzalutamide-resistant prostate cancer cells. (**A**–**D**) LNCaP, LNCaP-ENR, MR49F prostate cancer cells and non-cancer cells (HFF, astrocytes) were plated in 60 mm diameter plates (~300,000 cells per plate) and treated with varying doses of MK591 for 72 hours. Photographs were taken with a digital camera attached to a Leica microscope at x400, and cell viability was measured by MTS/PES assay. (**E**) Protein levels of 5-Lox were detected by Western blot. The full-length blots are presented in Supplementary Fig. 1. (**F**,**G**) Morphology and viability of LNCaP-ENR cells treated with lentiviral 5-Lox shRNA.

We observed that treatment with MK591 severely alters the morphology (Fig. 3A,B), and decreases the viability (Fig. 3D), of enzalutamide-resistant LNCaP-ENR and MR49F prostate cancer cells in a clear dose-dependent manner. Interestingly, MK591 does not affect the morphology and viability of normal, non-cancer cells, such as human fore-skin fibroblasts (HFF) or astrocytes in the same experimental conditions (Fig. 3C,D). To understand the molecular basis of this differential sensitivity to 5-Lox inhibitors, we found that in contrast to ERPC cells which express high-levels of 5-Lox, the expression of 5-Lox in normal fibroblasts or astrocytes is undetectable (Fig. 3E). Similarly, morphological alterations and a decrease in viability was also found when the ERPC cells were treated with 5-Lox shRNA, confirming a critical role of 5-Lox in the viability of ERPC cells (Fig. 3F,G). These novel findings suggest that inhibition of 5-Lox may turn out to be a useful strategy to selectively target ERPC cells without harming normal, non-cancer other body cells which do not express 5-Lox under normal conditions. *Note:* A noticeable increase in 5-Lox protein level and resistance to MK591 were observed in ERPC cells compared to the parental LNCaP cells (Fig. 3D,E).

### MK591 destabilizes mitochondria and induces apoptosis in Enzalutamide-resistant prostate cancer cells

**Figure 4:**
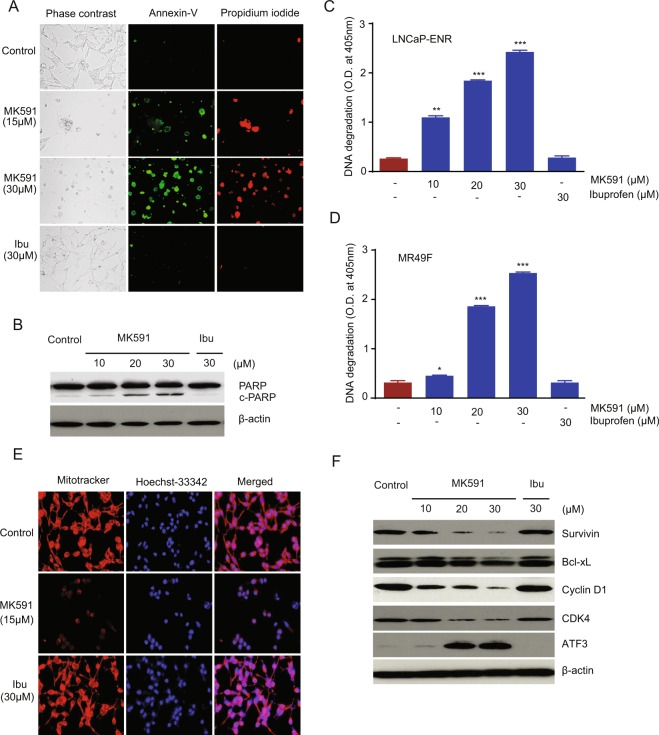
Induction of apoptosis in Enzalutamide-resistant prostate cancer cells by MK591. (**A**) LNCaP-ENR cells were plated in 60 mm diameter plates and treated with drugs as indicated for 24 hours. Externalization of phosphatidylserine was measured by binding with Annexin-V in isotonic buffer using a kit from BD Biosciences. The DNA-binding compound, Propidium iodide, was used to check for loss of membrane permeability, a characteristic of dead cells. (**B**) Characteristic cleavage of PARP after drug treatment for 24 hours. Beta actin was used as loading control. (**C**,**D**) Degradation of chromatin DNA to nucleosomal fragments in LNCaP-ENR and MR49F cells respectively by Cell Death ELISA, 24 hours after drug treatment. (**E**) Destabilization of mitochondria by MK591 as detected by the mitochondrial dye (mitotracker red) 24 hours after treatment. The DNA-binding dye, Hoechst-33342, was used to show the location of the nuclei. (**F**) Effect of MK591 on levels of cell cycle and apoptosis-regulating proteins are shown. Note: Pro-survival proteins (survivin, Bcl-xL, Cyclin D1, CDK4) are degraded while ATF3, a proapoptotic protein, was found to be increased with MK591 treatment. The full-length blots are presented in Supplementary Fig. 1.

Based on the morphological alterations of ERPC cells with 5-Lox inhibition, we wanted to determine whether these cells are undergoing apoptotic death. By analyzing standard mechanisms of apoptosis, we observed that ERPC cells show distinctly positive binding with annexin-V when treated with MK591, which suggests for externalization of phosphatidylserine to the outer layer of plasma membrane (Fig. 4A). Characteristic cleavage of PARP (poly-ADP ribose polymerase) by activated caspase is another indicator of apoptosis, which was also observed in ERPC cells after MK591 treatment (Fig. 4B). Moreover, we found that MK591 triggered degradation of chromatin DNA to nucleosomal fragments in LNCaP-ENR and MR49F (Fig. 4C,D) cells, confirming that inhibition of 5-Lox kills the ERPC cells via triggering of apoptosis. We also observed that MK591 induces mitochondrial permeability transition in ERPC cells, which is indicated by the loss of retention of the permeability-sensitive dye, mitotracker-red (Fig. 4E). Finally, we observed dramatic changes in the protein levels of cell survival- and apoptosis-regulators, such as survivin, Bcl-xL, cyclin D1, CDK4, and ATF3 (Fig. 4F).

### Inhibition of 5-Lox kills Enzalutamide-resistant prostate cancer cells via caspase-mediated apoptosis

**Figure 5:**
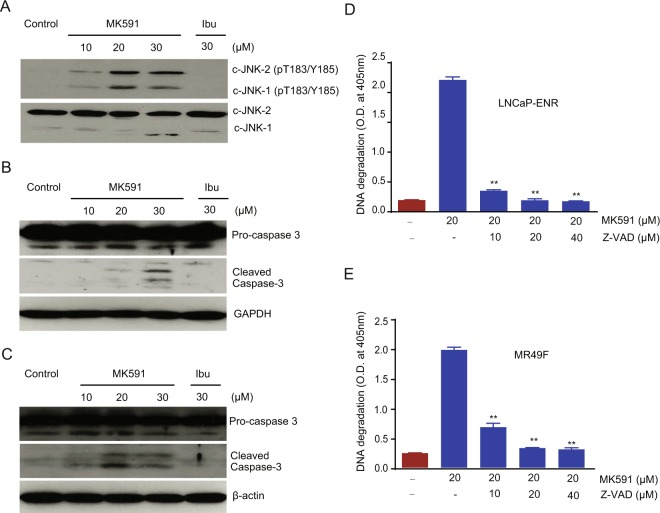
Activation and role of caspase in MK591-induced apoptosis in enzalutamide-resistant prostate cancer cells. (**A**) LNCaP-ENR cells (~300,000 per plate) were plated in 60 mm diameter plates and treated with drugs as indicated for 24 hours. Phosphorylation/activation of c-Jun N-terminal Kinase (c-JNK) was detected by Western blot using phospho-specific c-JNK antibody. (**B**,**C**) LNCaP-ENR and MR49F cells respectively, were treated with varying doses of MK591 and activation/cleavage of caspase-3 was detected by Western blot. The full-length blots are presented in Supplementary Fig. 1. (**D**,**E**) MK591-induced apoptotic DNA degradation (in LNCaP-ENR and MR49F cells, respectively) were strongly inhibited by Z-VAD, a general caspase inhibitor in a dose-dependent manner.

To explore the underlying mechanism in 5-Lox inhibition-induced apoptosis in ERPC cells, we analyzed effect of MK591 on Jun N-terminal kinases (JNKs) which play a critical role in apoptotic pathways^28^. We found that inhibition of 5-Lox triggers rapid and robust activation of the c-JNK in a dose-dependent manner in LNCaP-ENR cells (Fig. 5A). Both caspase-dependent and caspase-independent apoptotic cell death processes are known^29,30^. Previously, we have addressed the mechanism behind 5-Lox inhibition-induced apoptosis-triggering in prostate cancer cells which revealed involvement of caspase activation^31–34^. Here, we observed that treatment with MK591 induces activation of caspase 3 in LNCaP-ENR and MR49F cells (Fig. 5B,C). Moreover, it was found that the MK591 treatment-induced apoptosis in ERPC is inhibited when the cells were pretreated with a caspase inhibitor, Z-VAD-FMK, suggesting that 5-Lox inhibition-induced apoptosis in ERPC cells is dependent on caspase activity (Fig. 5D,E). Interestingly, cells treated with ibuprofen (an inhibitor of cyclooxygenase) did not show any signs of apoptotic features, suggesting that the effect of 5-Lox inhibition to induce apoptosis in ERPC cells is highly selective.

### Inhibition of 5-Lox blocks invasion and soft-agar colony formation by LNCaP-ENR and MR49F prostate cancer cells *in vitro*

**Figure 6:**
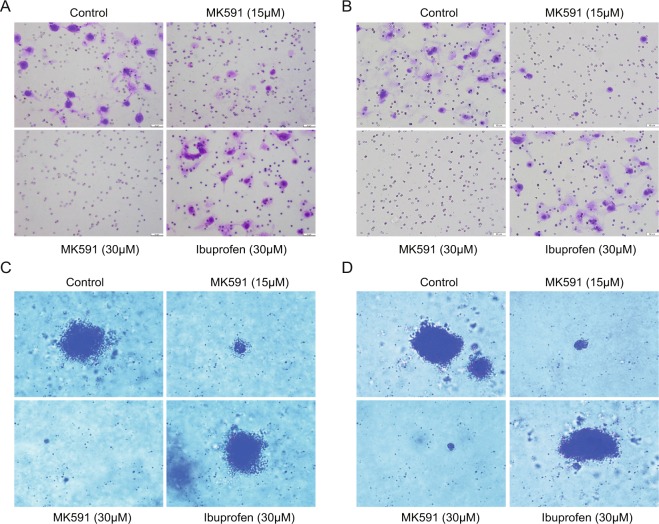
Effects of MK591 on invasion and soft-agar colony formation by enzalutamide-resistant prostate cancer cells. (**A**,**B**) Effect of MK591 on *in vitro* invasive capability of LNCaP-ENR and MR49F cells were measured using extracellular matrix-coated transwell chambers as described in the “Methods” section. After incubation, cells were fixed in methanol and stained with crystal violet. Pictures were taken with a Leica microscope at x200. (**C**,**D**) Effects of MK591 on soft-agar colony formation by LNCaP-ENR and MR49F cells are shown. Cells were plated on 0.3% soft-agar in RPMI medium and treated with inhibitors as indicated. Fresh media and inhibitors were added twice a week. Plates were incubated at 37 °C in the CO_2_ incubator for three weeks. At the end of incubation period, cells were stained with 0.025% crystal-violet, and colonies were counted, pictures taken with a microscope at x150. Note: Ibuprofen was used as a control which showed no effect on invasion or colony formation.

We found that treatment with MK591 at sub-lethal dose (10–20 μM) dramatically decreases the *in vitro* invasion through extracellular matrix by both LNCaP-ENR and MR49F ERPC cells (Fig. 6A,B). Moreover, we found that MK591 completely blocks the anchorage-independent colony-forming abilities of these ERPC cells on soft-agar (Fig. 6C,D). Thus, 5-Lox inhibitor drugs can stop the invasion and recolonization which are characteristics of advanced cancer phenotype. Interestingly, ibuprofen which was used in parallel experiments was found to be completely ineffective to block invasion or soft-agar colony formation by the ERPC cells, which suggests for a highly selective effect of 5-Lox inhibition in these processes.

## Discussion

One of the most remarkable genetic events that happens during transition of prostate cancer from an androgen-dependent to an androgen-independent state, has been found to be the upregulation of the c-Myc oncogene^8–12^. c-Myc has been frequently found to be overexpressed in androgen-independent tumors as well as in tumor-derived cells, and it plays a vital role in the survival, proliferation and metastatic abilities of prostate cancer cells. When the androgen-sensitive LNCaP prostate cancer cells are chronically treated with enzalutamide to make resistant cells (LNCaP-ENR), they show overexpression of c-Myc both at the mRNA as well as protein levels (Fig. 1A–D). Interestingly, the LNCaP-ENR cells showed decreased levels of both AR and its downstream targets, PSA and NKX3.1, suggesting that the LNCaP-ENR cells developed resistance to enzalutamide without involving reactivation of the AR axis. This finding may have profound implications as a new therapeutic approach because it is extremely difficult to control growth of prostate cancer by targeting the AR-signaling axis, which at the same time, also suggests for the existence and/or development of additional survival mechanisms which help prostate cancer cells survive and grow without involvement of the AR-signaling. We also found that a noticeable increase in the protein levels of c-Myc and targets takes place with enzalutamide treatment in short-term culture (Fig. 1E). Moreover, we found that 10058-F4, an inhibitor of c-Myc, decreases the viability of the enzalutamide-resistant LNCaP-ENR prostate cancer cells (Fig. 1F) which suggests that the ERPC cells are dependent on c-Myc for their survival and growth. Recently, we reported that inhibition of 5-Lox activity down-regulates c-Myc in prostate cancer cells, but not in normal, non-cancer cells, suggesting a selective role of 5-Lox-mediated metabolism of arachidonic acid (a common dietary omega-6 fatty acid) in the regulation of c-Myc in cancer cells^17,18^. Since c-Myc plays an important role in ERPC phenotype, we wanted to test the hypothesis whether 5-Lox plays any role in the viability and growth characteristics of advanced, ERPC cells, which may open up a new avenue to overcome enzalutamide-resistance.

We observed for the first time that inhibition of 5-Lox by shRNA or by chemical inhibitor, MK591, strongly downregulates the mRNA and protein levels as well as transcriptional activity of c-Myc in the enzalutamide-resistant prostate cancer cells (Fig. 2). These findings indicate that c-Myc in ERPC cells is tightly regulated by 5-Lox activity and suggest that the oncogenic action of c-Myc in these cells can be controlled by suitable 5-Lox inhibitors. We also found that MK591 kills the enzalutamide-resistant LNCaP-ENR and MR49F cells in a clear dose-dependent manner suggesting that the 5-Lox activity plays an essential role in the survival of these therapy-resistant cells (Fig. 3A–D). Interestingly MK591 did not affect normal, non-cancer cells, such as the mouse brain cells (astrocytes) or human fore-skin fibroblasts (HFF) in the same experimental conditions, documenting a strong cancer-specific action of this agent. Further analysis revealed that in contrast to ERPC cells, which express high-levels of 5-Lox, the expression of 5-Lox in normal, non-cancer cells was undetectable (Fig. 3E). A role of 5-Lox in the viability of ERPC cells was also observed using 5-Lox gene-specific shRNA (Fig. 3F,G). This feature has far-reaching implications for development of a new therapy against ERPC, because under normal conditions 5-Lox is not expressed in non-immune parenchyma cells unless chronic inflammatory condition occurs, such as in asthma, arthritis and some types of cancer. Moreover, though 5-Lox gene is normally expressed in inflammatory cells (neutrophils, eosinophils, basophils), the 5-Lox protein is maintained in an inactive state which needs further stimulation by a calcium surge and ATP to transiently gain its catalytic activity^35,36^. Thus, our novel findings suggest that 5-Lox is an emerging, very promising molecular target for development of a new therapy against ERPC which is currently incurable primarily due to the lack of proper understanding about suitable molecular target(s) to selectively and effectively attack and kill the ERPC cells.

Based on the observed morphological alterations in enzalutamide-resistant cells upon MK591 treatment, we suspected that these cells are presumably undergoing apoptosis. We performed a set of experiments and found that MK591 treatment induces externalization of phosphatidylserine as measured by binding with annexin-V and characteristic cleavage of PARP (Fig. 4A,B). In addition, the MK591-treated cells show distinctive degradation of chromatin DNA to nucleosomal fragments, which is the characteristic feature of advanced stage of apoptotic cell death (Fig. 4C,D). Moreover, we observed that MK591 induces mitochondrial permeability transition in ERPC cells (Fig. 4E). These findings indicate that MK591 inhibits c-Myc function and kills enzalutamide-resistant prostate cancer cells via induction of apoptosis. By analyzing several apoptosis and cell cycle-regulatory proteins, we found that treatment with MK591 decreased the protein levels of survivin, Bcl-xL, cyclin D1 and CDK4, which are pro-survival, and increased the levels of ATF3 which is pro-apoptotic (Fig. 4F).

The stress-activated c-Jun N-terminal Kinase (c-JNK) is activated and plays a role in the apoptosis process in a variety of cancer cells. Activation and role of c-JNK in caspase-mediated apoptosis have been reported in prostate cancer cells^28^. Our observation of a rapid, dose-dependent activation of c-JNK by Mk591 in enzalutamide-resistant prostate cancer cells suggests that c-JNK may play a role in the MK591-induced apoptosis in these cells (Fig. 5A). We further wanted to study the nature of apoptosis in ERPC cells by MK591 because both caspase-dependent and –independent mechanisms of apoptosis have been characterized. We found that treatment with MK591 generated characteristic cleavage products of pro-caspase 3, indicating that this type of apoptosis may involve the activity of caspase (Fig. 5B,C). We confirmed this by using specific caspase-3 inhibitors which strongly inhibited apoptotic DNA degradation, documenting that MK591-induced apoptosis in ERPC cells is caspase-dependent (Fig. 5D,E).

Therapy-resistant cancer cells develop extraordinary ability to invade surrounding tissues, move to distant sites and recolonize to generate metastatic nodules which ends up with debilitating conditions of patients. Since, majority of the deaths due to prostate cancer happen because of metastasis to distant organs, such lymph nodes and bones, it is appropriate to find agents that could be used either individually or in combination with other agents to prevent metastatic progression of prostate cancer. Thus, drugs that can stop the invasion and/or recolonization are useful to delay onset of advanced cancer phenotypes. Since, c-Myc is overexpressed in advanced stages of prostate cancer and is intimately associated with castration-resistant, metastatic phenotype, we wanted to explore the effects of MK591 in *in vitro* invasion and anchorage-independent colony-formation by ERPC cells. In our study, we found that MK591 strongly inhibits *in vitro* matrigel invasion (Fig. 6A,B), and soft-agar colony formation (Fig. 6C,D), by both LNCaP-ENR and MR49F cells at sub-lethal doses. Our *in vitro* finding of a dramatic reduction of invasive as well soft-agar colony forming abilities of LNCaP-ENR as well as MR49F cells by sub-lethal doses of MK591 suggests that the aggressive and metastatic tumor-forming ability of ERPC cells could be effectively controlled by MK591 or similar other suitable *in vivo*-effective 5-Lox inhibitors.

**Figure 7:**
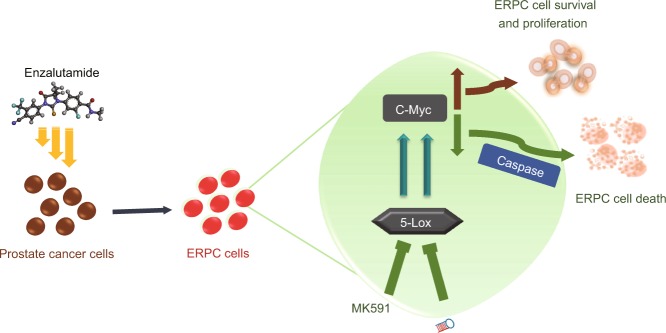
A model depicting regulation of the viability and death in enzalutamide-resistant prostate cancer cells by 5-Lox, as revealed by pharmacological and genetic targeting.

Anti-androgenic therapies are commonly used in the clinic which extends life-span but castration-resistant disease invariably develops. More than 90% of the CRPC patients end up with bone metastasis causing excruciating pain and suffering^37^. While Enzalutamide, an FDA-approved inhibitor of androgen receptor, improves survival and quality of life in CRPC patients, which highlights the success of targeting the AR axis in CRPC, these therapies are not curative because development of resistant disease is inevitable, and because the resistant cells are not killed effectively by available other clinical regimens^2–5^. We have repeatedly found that the 5-Lox activity plays an important role in the survival of advanced prostate cancer cells, including prostate cancer stem cells^18,31–34,38,39^. Since, c-Myc is heavily expressed and plays a pivotal role in enzalutamide-resistant prostate cancer and is tightly regulated by 5-Lox, our findings indicate that inhibition of 5-Lox by MK591 may turn out to be an effective strategy to overcome enzalutamide-resistance in prostate cancer via downregulation of the c-Myc oncogene and selective induction apoptosis in ERPC cells (Fig. 7).

## Methods

### Cell culture and reagents

LNCaP human prostate cancer cells and human fore-skin fibroblasts (HFF) were purchased from American Type Culture Collection (Manassas, VA). The enzalutamide-resistant LNCaP-ENR cells were generated by exposing LNCaP cells to an increasing concentration of enzalutamide over 6 months. The enzalutamide-resistant MR49F cells were provided by Dr. Martin Gleave (Vancouver, Canada). These cells were developed by repeated (3-times) transplantation of LNCaP cells into castrated mice, followed by isolation of tumor-derived cells and maintaining them in long-term culture in the presence of 10 μM enzalutamide^21^. Cells were grown either in RPMI medium 1640 or DMEM (Invitrogen, Carlsbad, CA). All the media were supplemented with 10% FBS and antibiotics. Antibodies against c-Myc and survivin were purchased from R and D Systems (Minneapolis, MN), and antibodies against, Bcl-xL, cyclin D1, CDK4, ATF3, and GAPDH were from Santa Cruz Biotechnology (Santa Cruz, CA). Polyclonal anti-5-Lox antibody was from ProteinTech (Chicago, IL), and anti-beta-actin antibody was purchased from Sigma Chemical CO (St. Louis, MO). MK591 was obtained as a generous gift from Dr. Robert N. Young (Merck-Frosst Centre for Therapeutic Research, Quebec, Canada).

### Cell viability assay

Cell viability was measured by MTS/PES One Solution Cell Titer Assay (Promega Corp, Madison, WI) as described before^38,40^.

### Microscopy

Cells (~300,000) were plated overnight in RPMI medium 1640 supplemented with 10% FBS onto 60 mm diameter tissue culture plates (Falcon) and allowed to grow for 48 hours. On the day of experiment, the spent culture medium was replaced with 2 ml fresh RPMI medium and the cells were treated with inhibitors. Control cells were treated with solvent (DMSO). Photographs were taken with a Nikon digital camera attached to a LEICA microscope at x400. Image acquisition and data processing were done with a Dell computer attached to the microscope using Q-Capture 7 software.

### Real-Time quantitative PCR

Total RNA was extracted using RNeasy kit (Qiagen) and 1 µg of total RNA was used for cDNA synthesis using SuperScript III First-Strand kit (Invitrogen) according to the manufacturer’s instructions. PCR reaction mixture was prepared using gene specific TaqMan gene expression assay system (Applied Biosystems). qRT-PCR reactions were performed in triplicate using QuantStudio 6 Flex Real-Time fast PCR System (Applied Biosystems) and 2^−ΔΔCt^ values were used to calculate the relative expression level of the target genes compared to controls. GAPDH was used as a normalization control.

### Western blot

Cells (~300,000) were plated in 60 mm diameter plates and allowed to grow for 48 hours. The old medium was then replaced with 2 ml fresh RPMI medium and then the cells were treated with inhibitors. After treatment, cells were harvested, washed, and lysed in lysis buffer (50 mM HEPES buffer, pH 7.4, 150 mM NaCl, 1 mM EDTA, 1 mM orthovanadate, 10 mM sodium pyrophosphate, 10 mM sodium fluoride, 1% NP-40, and a cocktail of protease inhibitors). Proteins were separated by 12% SDS–PAGE and transferred to nitrocellulose membranes. Membranes were blocked with 5% nonfat-milk solution and blotted with appropriate primary antibody followed by peroxidase-labeled secondary antibody. Bands were visualized by enhanced chemiluminescence detection kit from Pierce Biotech (Rockford, IL) and analyzed with a densitometer using Kodak imaging software. To be accepted as valid, protein blots were analyzed at least in two independent experiments showing similar results.

### Luciferase assay

Cells were plated overnight and transfected with lentiviral E-box-luciferase constructs (>90% cells transfected), expanded, and re-plated in 96 well culture plates in triplicates. Cells were then treated with MK591 or control solvent for 24 hours, and the luciferase activity was measured by a luciferase assay kit from Promega Corporation (Madison, WI) as described earlier^17^. Ibuprofen was used as negative control in parallel assays.

### DNA-degradation assay

Cells (~300,000) were plated in 60 mm diameter plates and allowed to grow for 48 hours. The old medium was then replaced with 2 ml fresh RPMI medium and then the cells were treated with inhibitors for 24 hours. Drug-treated and control cells were lysed in lysis buffer for 60 minutes at 4 °C and aliquots of lysates were used for measuring DNA-degradation to nucleosomal fragments using an ELISA kit from Roche (St. Louis, MO) as reported before^38^. Ibuprofen was used as negative control for assay validation.

### Invasion assay

*In vitro* invasion assay was done using matrigel-coated Boyden transwell chambers from BD Biosciences. Transwells were soaked in 50 µl serum-free medium for 30 minutes at RT and then ~40,000 cells (in RPMI medium containing 0.1% BSA) were placed into the upper chambers. These chambers were then placed in 24 well plate (one per well) on top of 500 µl RPMI medium containing 3% fetal bovine serum as chemo-attractant. Inhibitors were added directly to the medium and mixed. Then the cells were incubated at 37 °C in the CO_2_ incubator for 16 hours. Non-invaded cells along with matrigel in the upper chambers were scraped with a cotton tipped applicator and then the membranes were fixed in methanol, stained with 0.025% crystal violet, and observed under a Leica microscope at x200.

### Soft-agar colony formation assay

Soft-agar colony formation assays were performed in six well plates by placing ~10,000 LNCaP cells in 0.5 ml of 0.3% soft-agar on top of a 2 ml base layer of 0.6% agar. Plates were allowed to settle and then the wells were covered with 2 ml fresh RPMI medium containing 10% FBS with or without inhibitors. Plates were incubated at 37 °C in the CO_2_ incubator for a maximum period of three weeks. Cell growth medium and inhibitors were exchanged every fourth day. At the end of incubation, cells were stained with 0.025% crystal violet and colonies were counted and photographed under a Leica microscope at x150.

### Statistical analysis

Statistical significance was assessed by two-way analysis of variance (ANOVA) or the two-tailed Students t-test and a value of <0.05 was defined as significant. Results are expressed as the mean ± standard error of the mean (S.E.M.) and are described in each figure legend when applied.

## Supplementary information

Supplementary information.
