# Supplementary material for: Pharmacological and genetic targeting of 5-lipoxygenase interrupts c-Myc oncogenic signaling and kills enzalutamide-resistant prostate cancer cells via apoptosis
Source: Sci Rep. 2020 Apr 20;10:6649. doi: 10.1038/s41598-020-62845-8 (PMC7171151; doi:10.1038/s41598-020-62845-8)
Supplement: Supplementary file 1 — Supplementary information. [file 41598_2020_62845_MOESM1_ESM.pdf]

# Pharmacological and genetic targeting of 5-lipoxygenase interrupts c-Myc oncogenic signaling and kills enzalutamide-resistant prostate cancer cells via apoptosis

Jitender Monga[1], Dhatchayini Subramani[1], Ajay Bharathan[1] and Jagadananda Ghosh[1, 2, *]

[1] Vattikuti Urology Institute, [2]Henry Ford Cancer Institute, Henry Ford Health System, Detroit, MI 48202, United States.

[*]Correspondence
jghosh1@hfhs.org

**Fig. 1D**

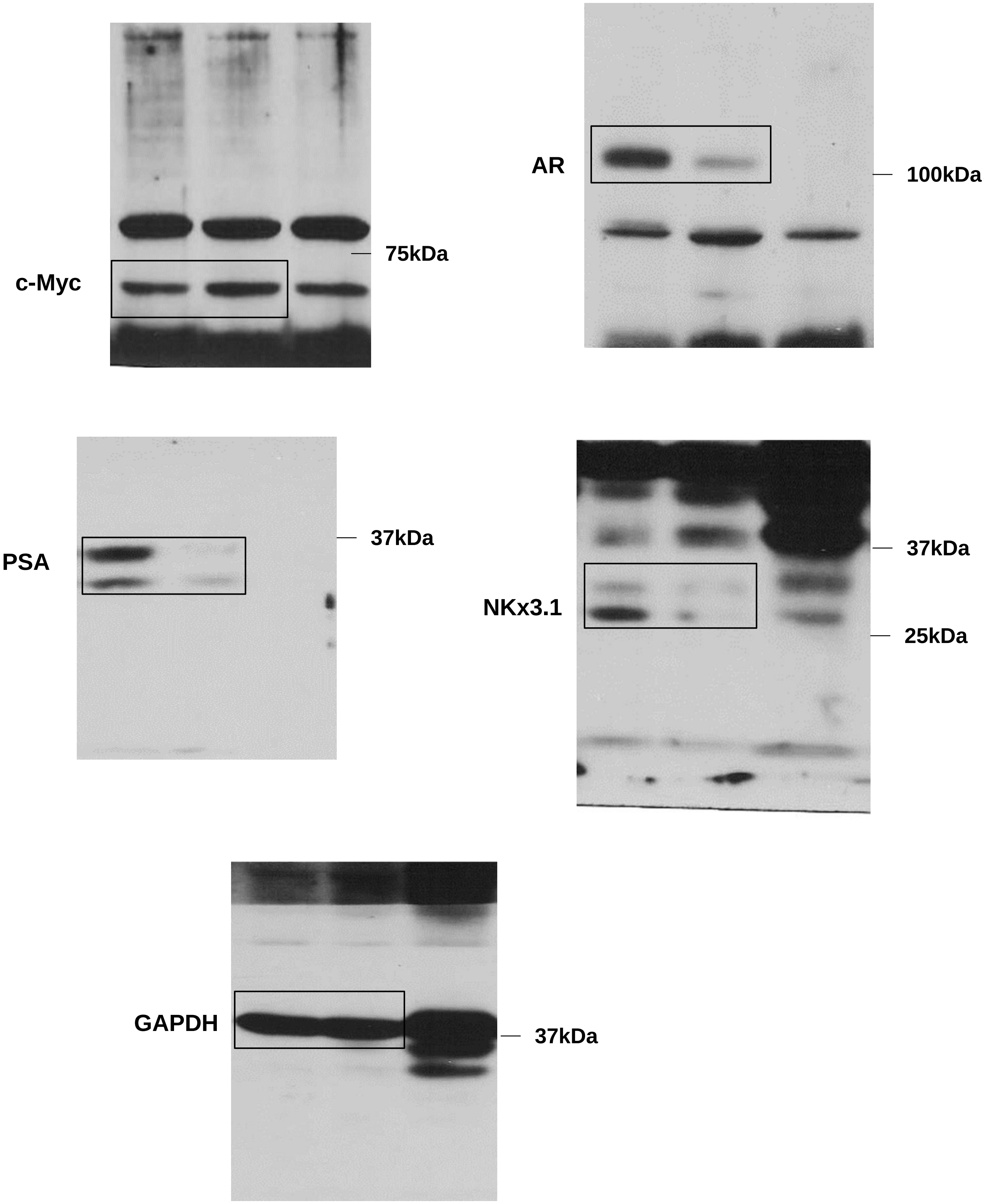


**Supplementary Figure 1.** Full-length scans of western blots. Black rectangles indicate regions used in figures.

## Fig. 1E

c-Myc

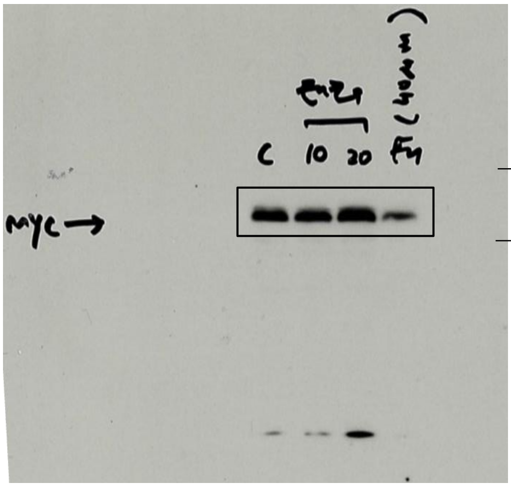


75kDa
50kDa

Cyclin D1

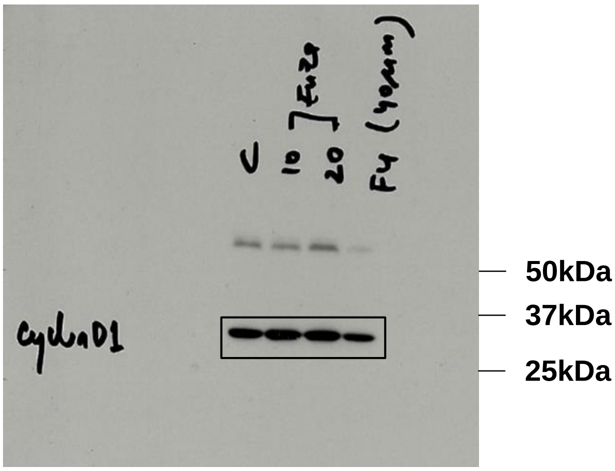


50kDa
37kDa
25kDa

β-actin

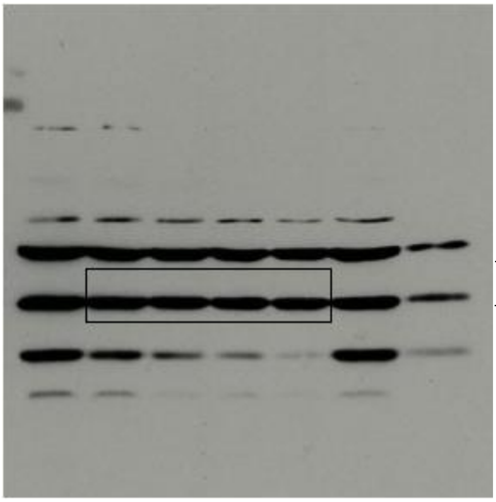

50kDa
37kDa

β-actin

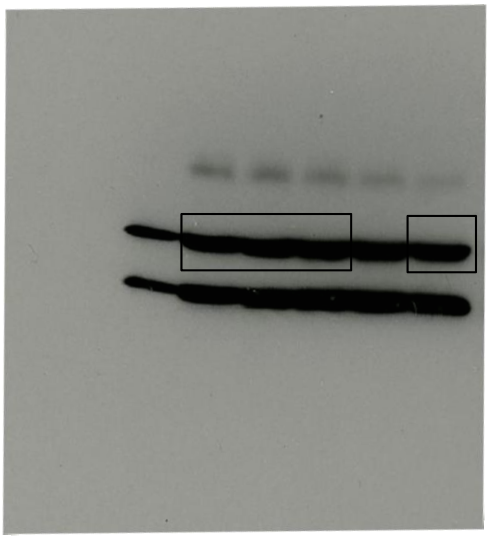

50kDa
37kDa

Aurora-B

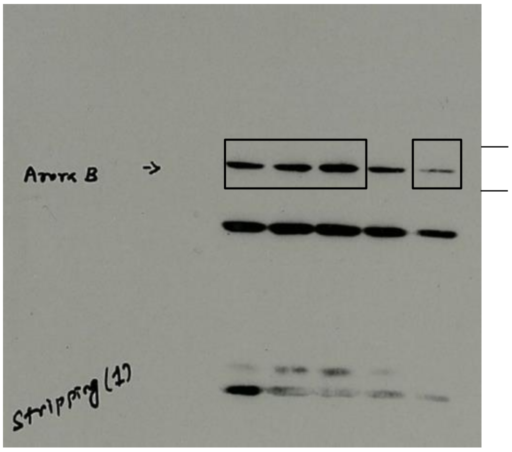


50kDa
37kDa

Supplementary Figure 1 continued

## Fig. 2A

c-Myc

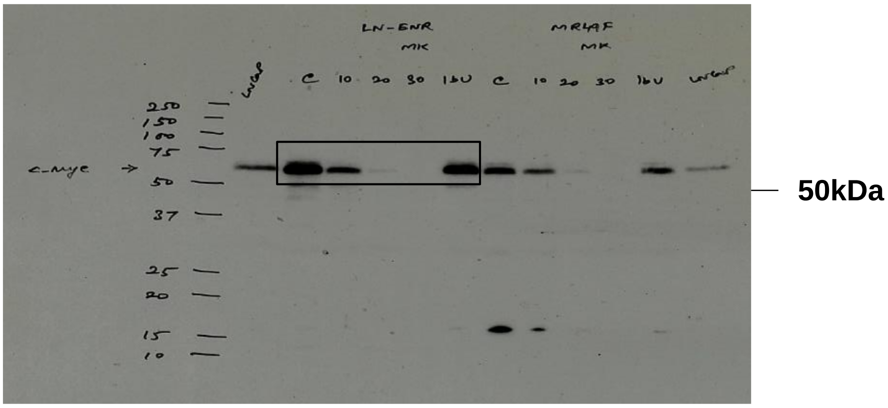


50kDa

β-actin

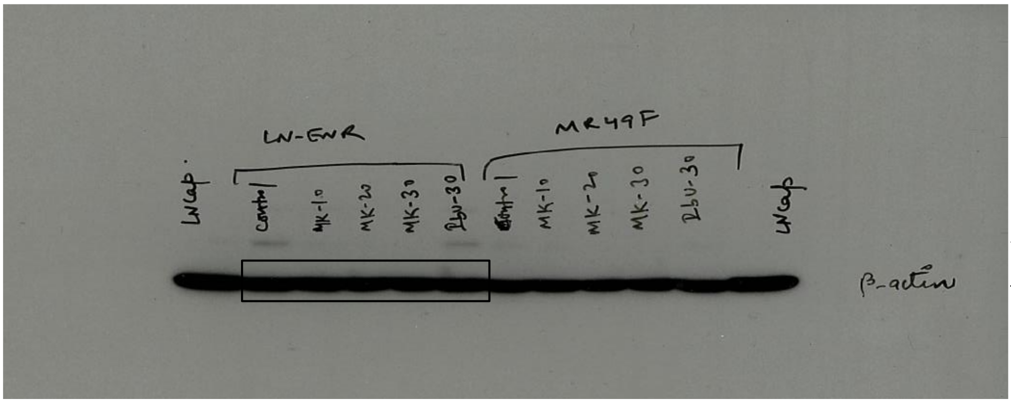


50kDa

37kDa

## Fig. 2B

c-Myc

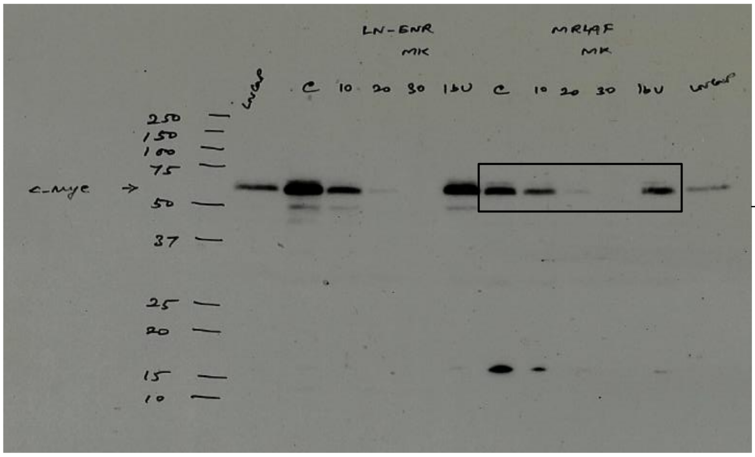


50kDa

β-actin

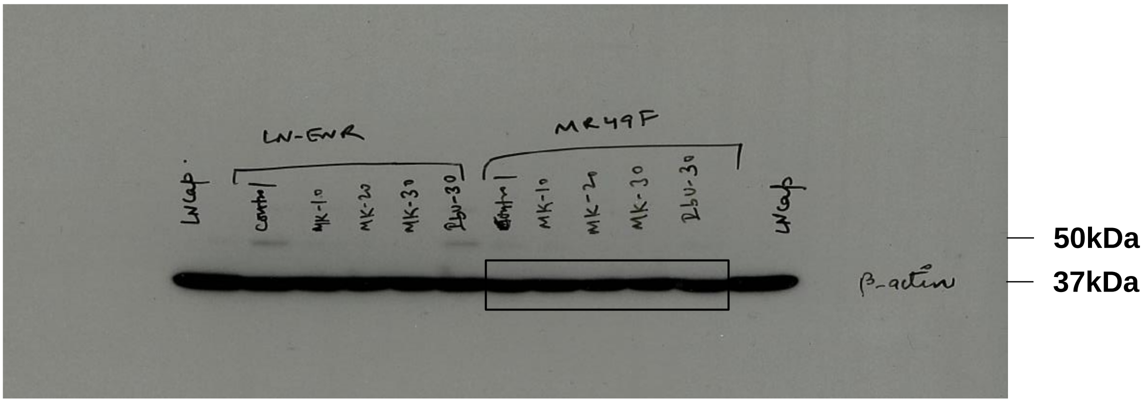


50kDa

37kDa

**Supplementary Figure 1 continued**

Fig. 2D

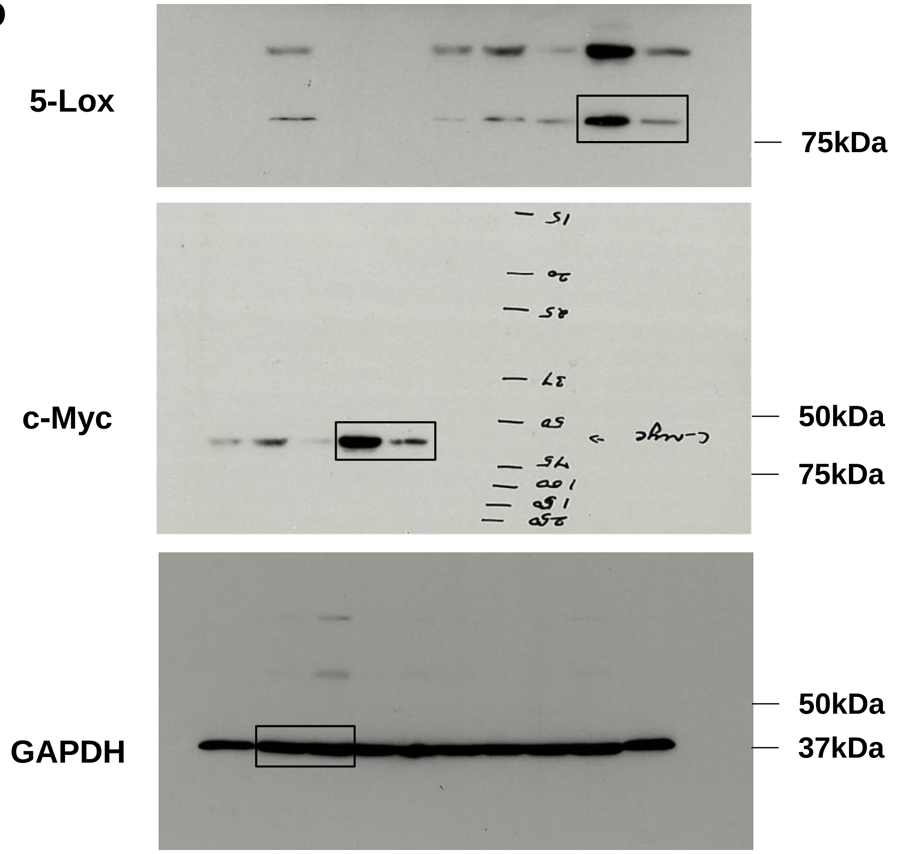


Fig. 2E

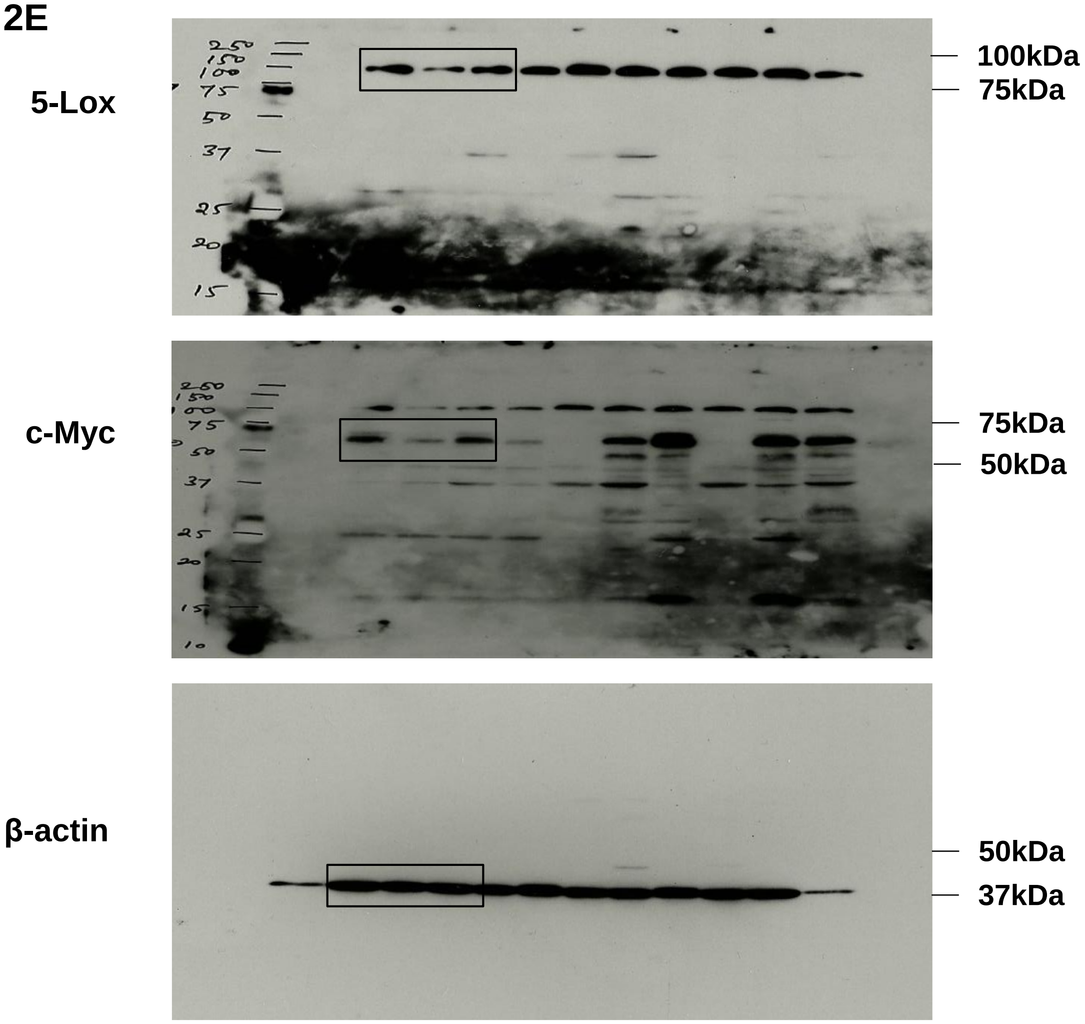


Supplementary Figure 1 continued

**Fig. 3E**

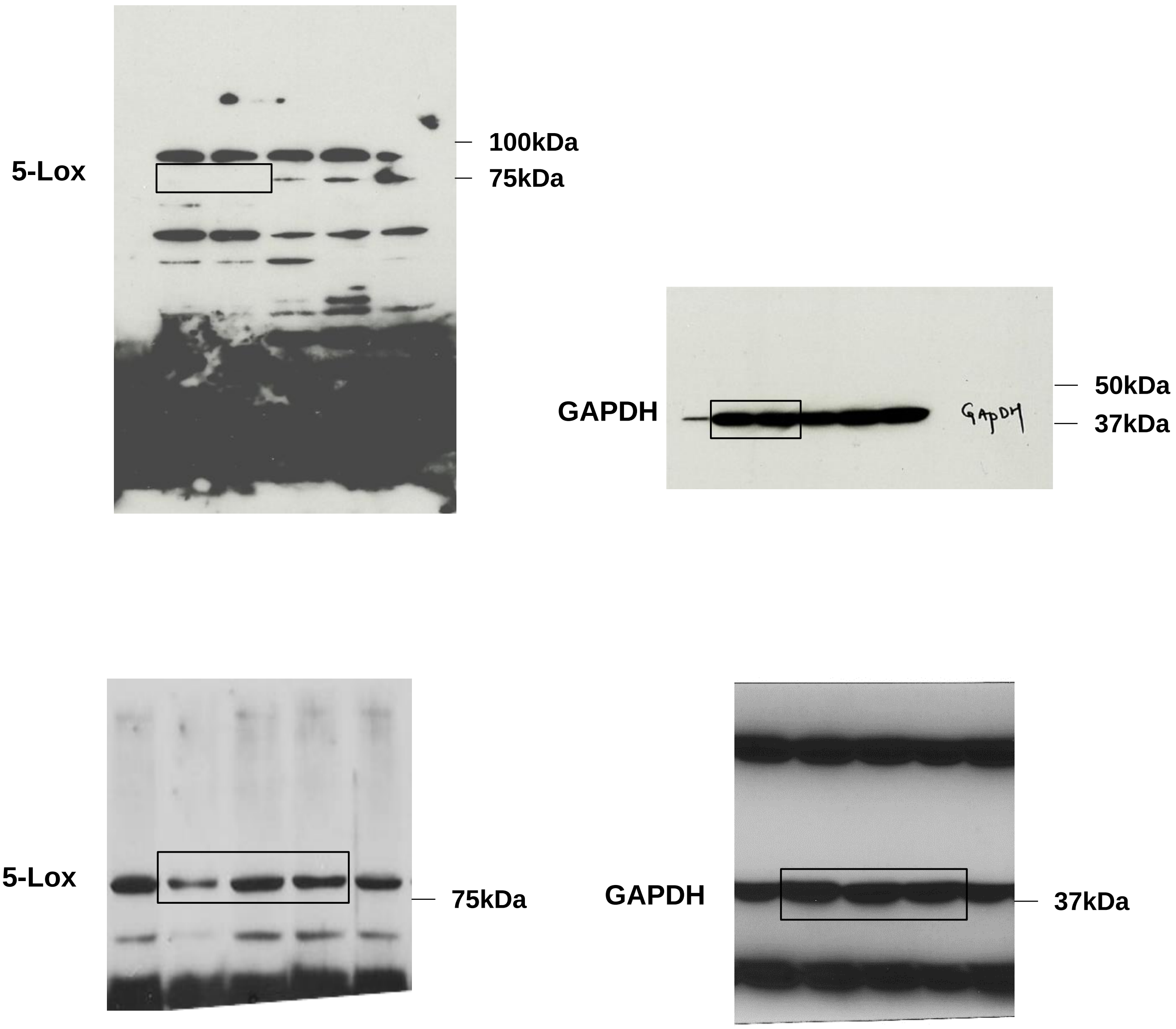


**Supplementary Figure 1 continued**

## Fig. 4B

PARP
c-PARP

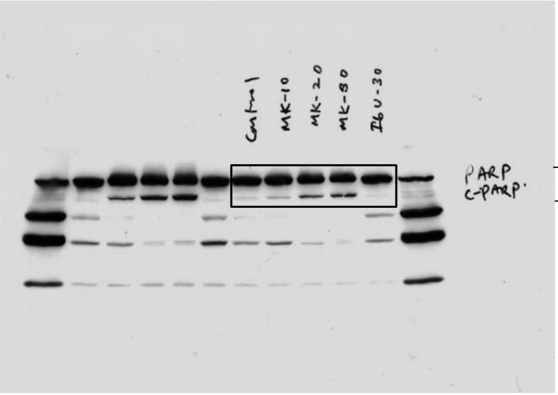


100kDa
75kDa

β-actin

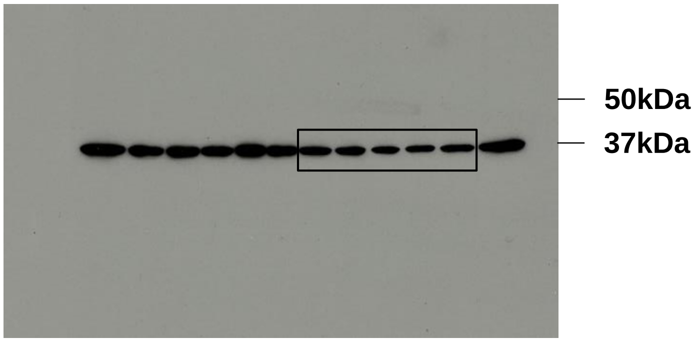

50kDa
37kDa

## Fig. 4F

Survivin

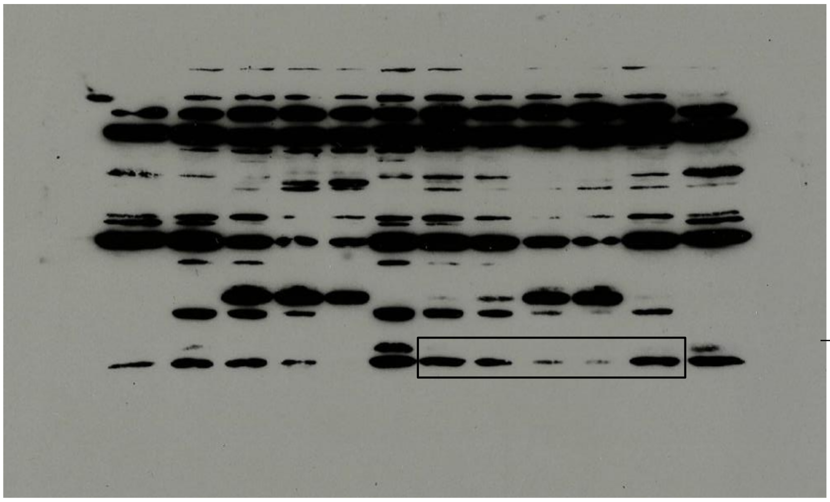

37kDa
25kDa

Fig. 4F continue

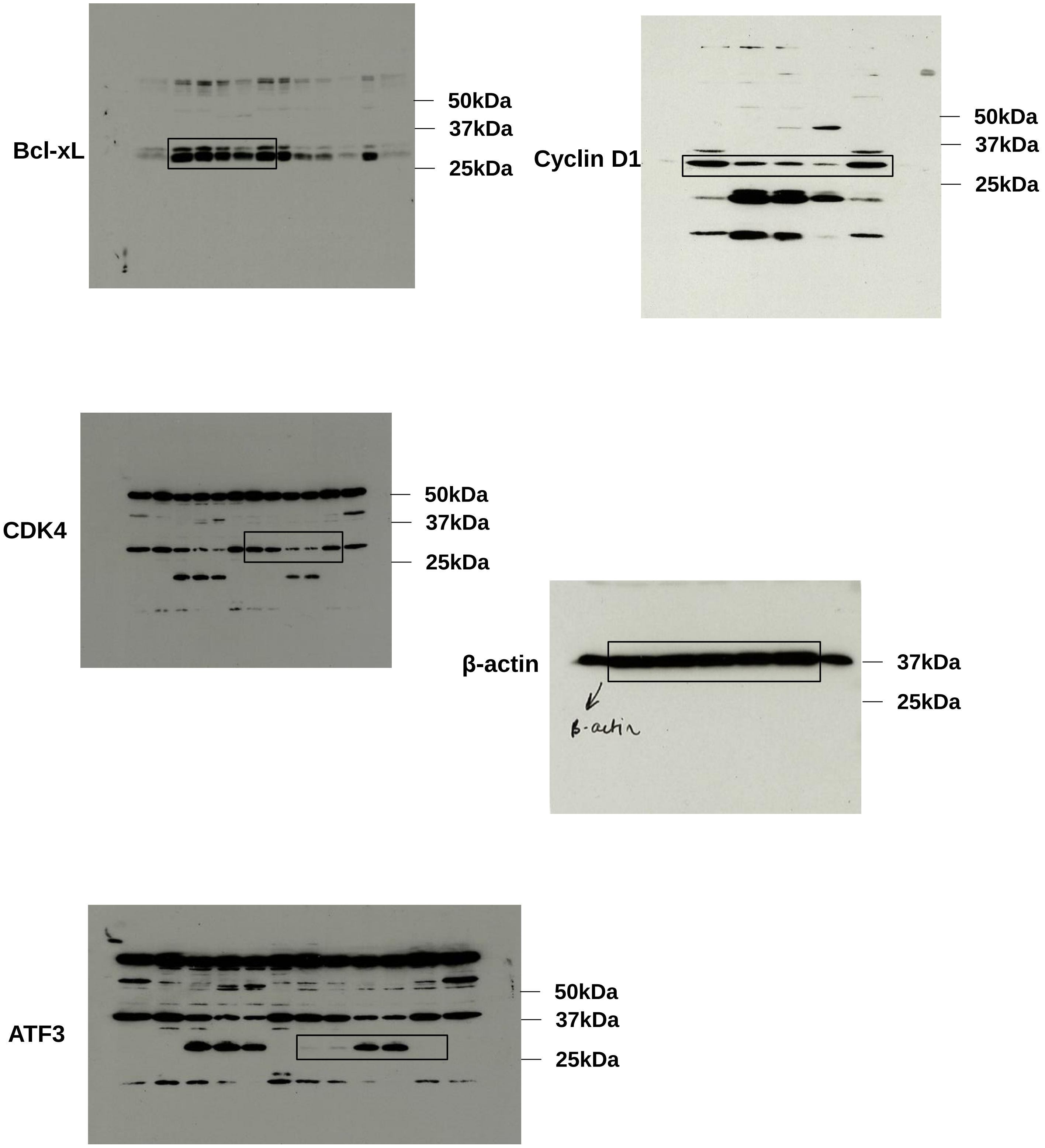

## Fig. 5A

c-JNK-2 (pT183/Y185)
c-JNK-1 (pT183/Y185)

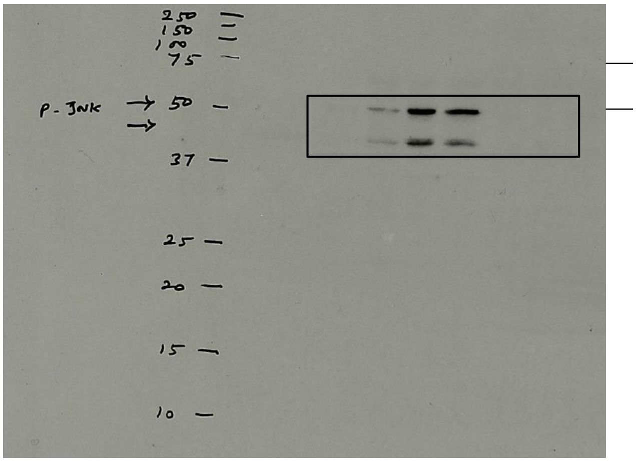


75kDa
50kDa

c-JNK-2
c-JNK-1

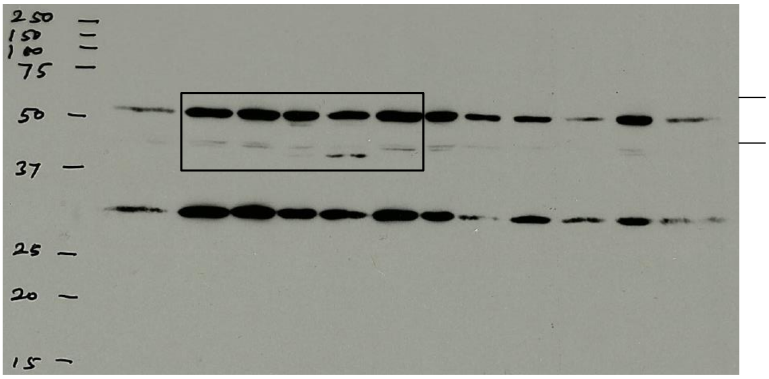


75kDa
50kDa

## Fig. 5B

Pro-caspase 3

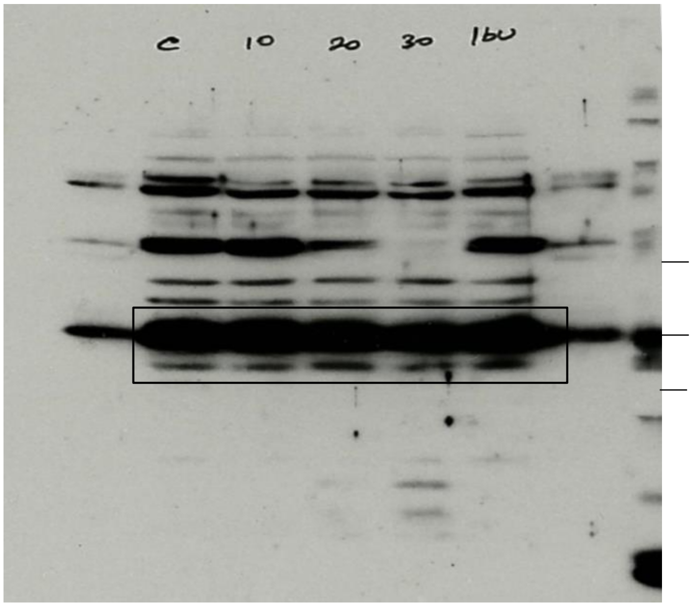


50kDa
37kDa
25kDa

## Fig. 5B continue

GAPDH

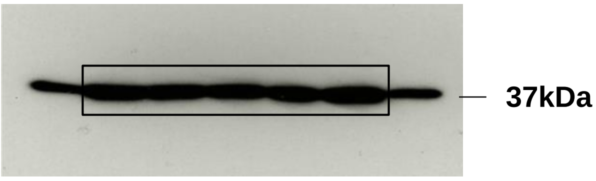

37kDa

Cleaved
Caspase-3

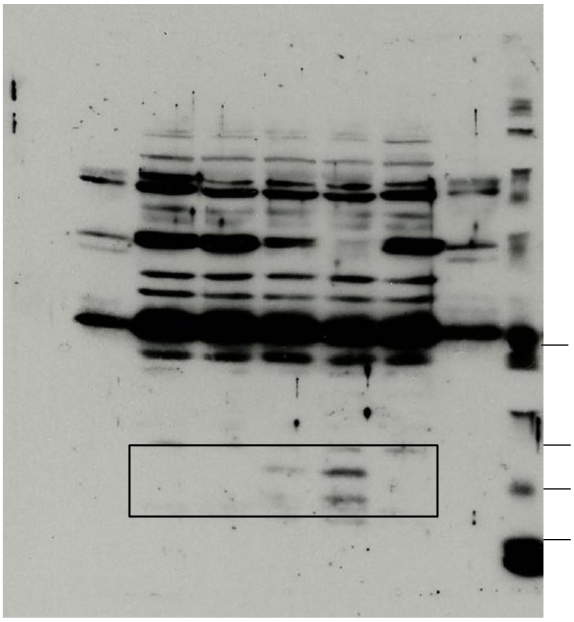

37kDa
25kDa
20kDa
15kDa

## Fig. 5C

Pro-caspase 3

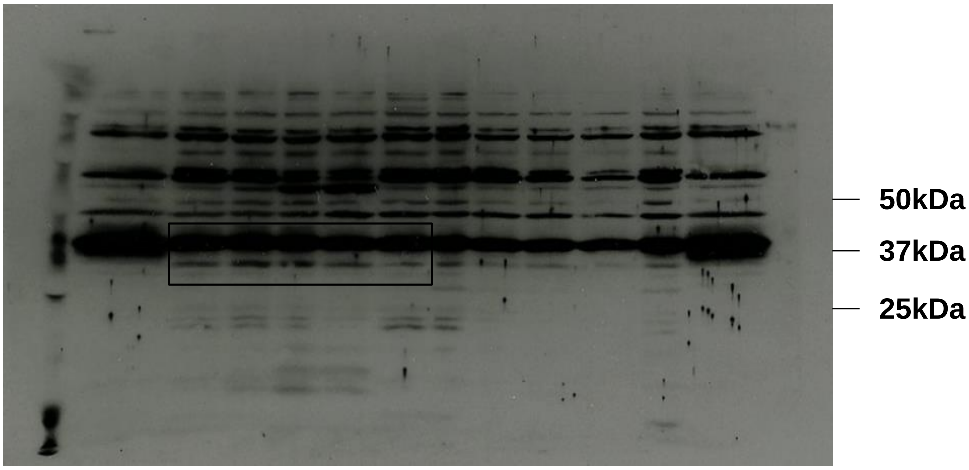

50kDa
37kDa
25kDa

Cleaved
Caspase-3

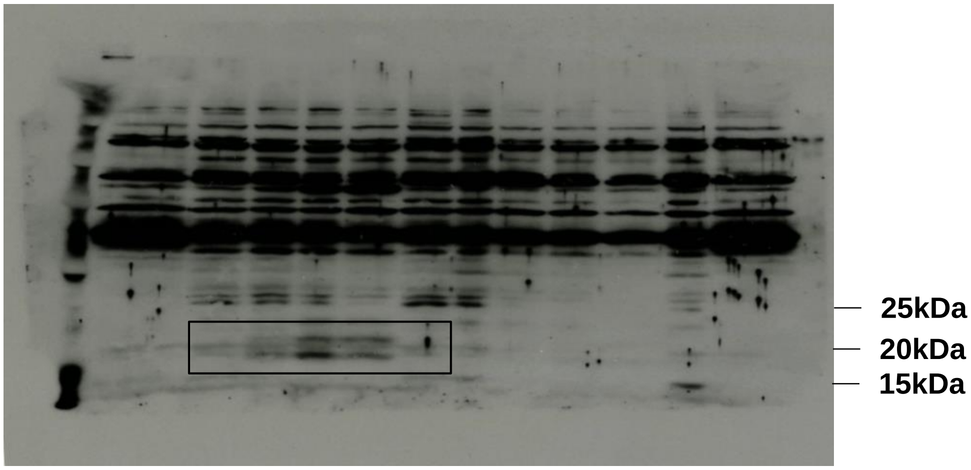

25kDa
20kDa
15kDa

Supplementary Figure 1 continued

## Fig. 5C continue

GAPDH

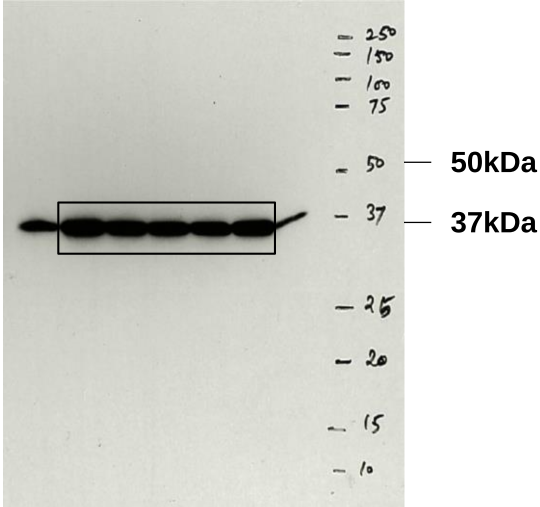


50kDa

37kDa

Supplementary Figure 1 continued
